# Supplementary material for: A sulfide-sensor and a sulfane sulfur-sensor collectively regulate sulfur-oxidation for feather degradation by Bacillus licheniformis
Source: Commun Biol. 2023 Feb 10;6:167. doi: 10.1038/s42003-023-04538-2 (PMC9918477; doi:10.1038/s42003-023-04538-2)
Supplement: Supplementary file 2 — Supplementary Information [file 42003_2023_4538_MOESM2_ESM.pdf]

Supplementary information for

A sulfide-sensor and a sulfane sulfur-sensor collectively regulate sulfur-oxidation for feather degradation by *Bacillus licheniformis*

Chao Tang[1#], Jingjing Li[1, 3#], Yuemeng Shen[1], Menghui Liu[1], Honglei Liu[1], Huaiwei Liu[1], Luying Xun[1, 2*], Yongzhen Xia[1*]

[1]State Key Laboratory of Microbial Technology, Shandong University, Qingdao, 266237, People's Republic of China

[2]School of Molecular Biosciences, Washington State University, Pullman, WA, 99164-7520, USA

[3]Institut für Mikrobiologie & Biotechnologie, Rheinische Friedrich-Wilhelms-Universität Bonn, Bonn, Germany

* To whom correspondence should be addressed to L. Xun. Tel: +1-509-335-2787; Email: luying_xun@vetmed.edu.edu; Correspondence may also be addressed to Y. Xia. Tel: +86-532-58631572; Email: xiayongzhen2002@sdu.edu.cn;

[#] These two authors contributed equally.

Including

Supplementary Figure 1 – Supplementary Figure 14

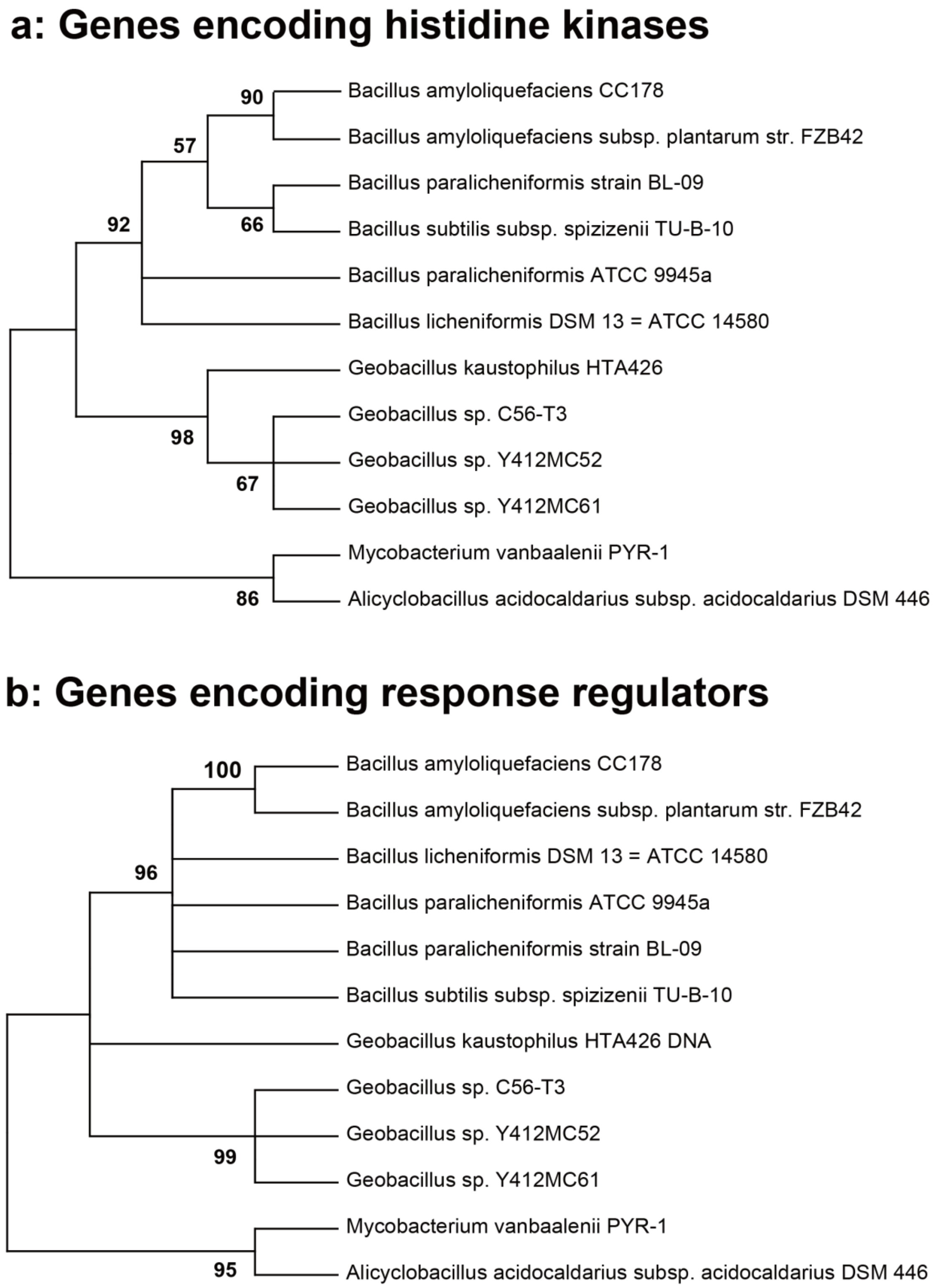

Supplementary Figure 1. The phylogenetic tree of 12 TCSs whose genes are located near *sqr-pdo* genes. These sequences were aligned by using ClustalW, and the trees were built by using MEGA X with the Maximum Likelihood method and Tamura-Nei model.

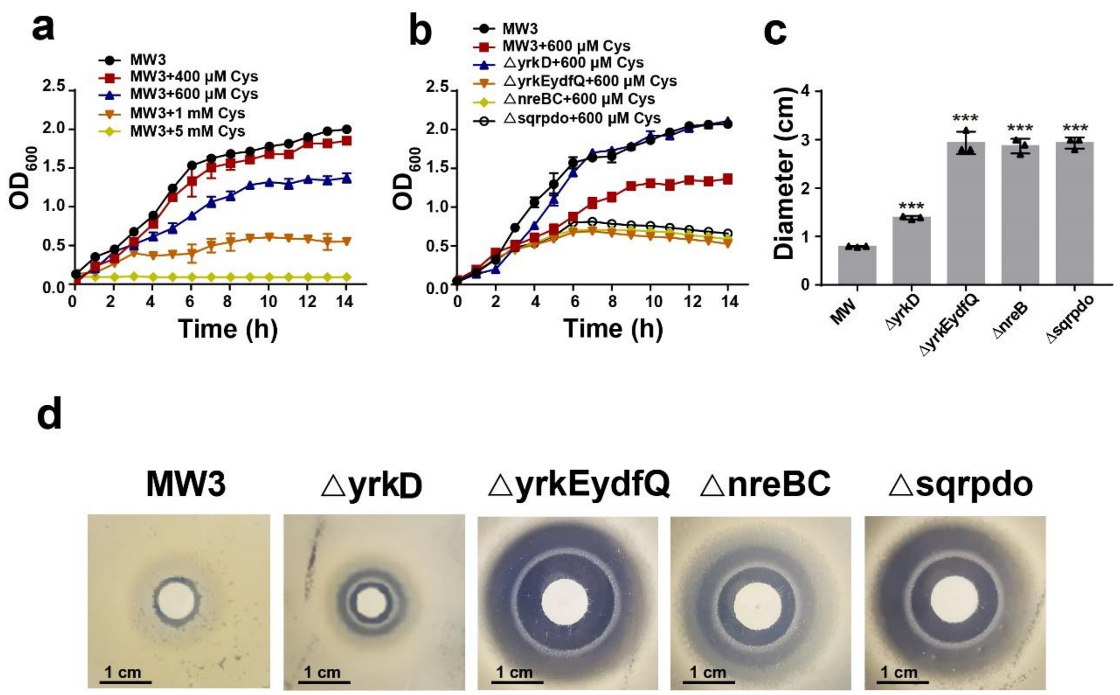

Supplementary Figure 2. The growth adaption of *B. licheniformis* MW3 to gradient concentrations of cysteine in LB medium or agar plates. a-b) *B. licheniformis* MW3 and its mutants were inoculated into LB medium with or without different concentrations of Cys at the initial OD_{600} of 0.1 and cultured in a 24-well cell culture plate with continuous shaking at 37°C. The OD_{600} values were recorded at defined time intervals. **c-d)** *B. licheniformis* MW3 and its mutants were spread on LB plates, and then 3 μL 8 M cysteine solution was dropped in the middle of the plates. After incubation for 24 hours, the sizes of the clear zones were recorded. Three replicates were done for each strain to obtain averages and standard deviations **(c)**, and the representative pictures were shown **(d)**. Scale bars – 1 cm. Three parallel experiments were performed to obtain the averages and standard derivations. Data are presented as mean ± SD. One-way ANOVA was performed to calculate the p-values with MW3 as the control, and asterisks indicate a statistically significant difference (***p <0.001). For **Supp Fig. 3d**, 3 parallel experiments were done. The results were

similar, and only the representative results were presented.

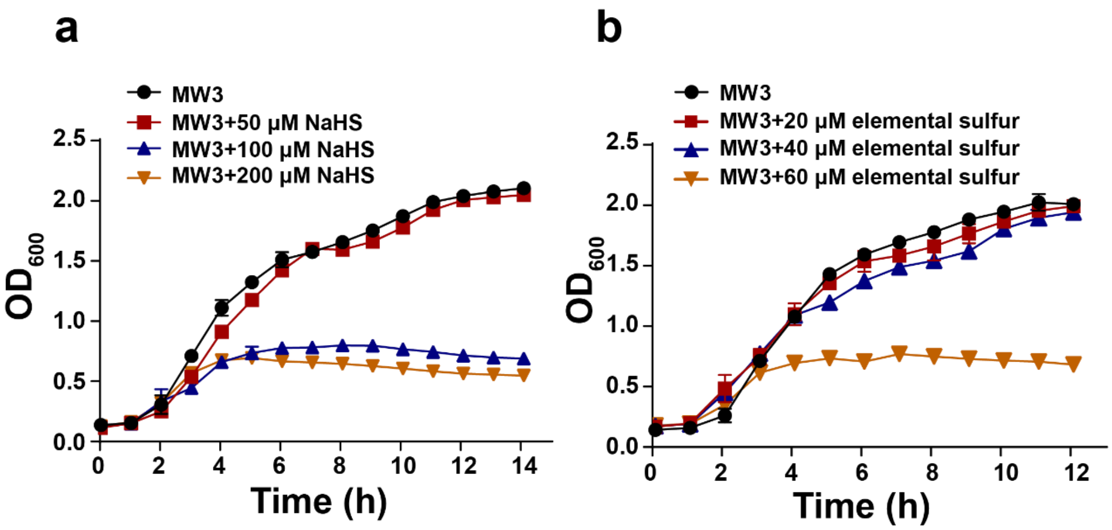

Supplementary Figure 3. The growth of *B. licheniformis* MW3 in gradient concentration of NaHS and elemental sulfur. a&b) *B. licheniformis* MW3 was inoculated into LB medium with or without different concentrations of NaHS and elemental sulfur at the initial OD_{600} of 0.1 and cultured in a 24-well cell culture plate with continuous shaking at 37°C. The OD_{600} values were recorded at defined time intervals. Three parallel experiments were performed to obtain the averages and standard derivations. Data are presented as mean ± SD.

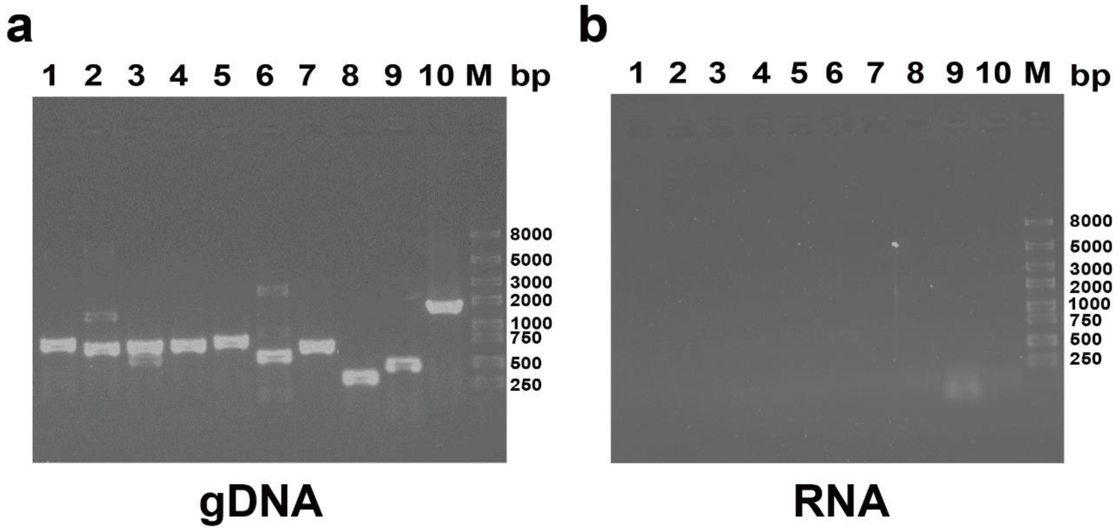

Supplementary Figure 4. The qPCR test with 10 pairs of primers to amplify gDNA and RNA isolated from the induced *B. licheniformis* MW3. They are the controls for the results shown in Figure 4a.

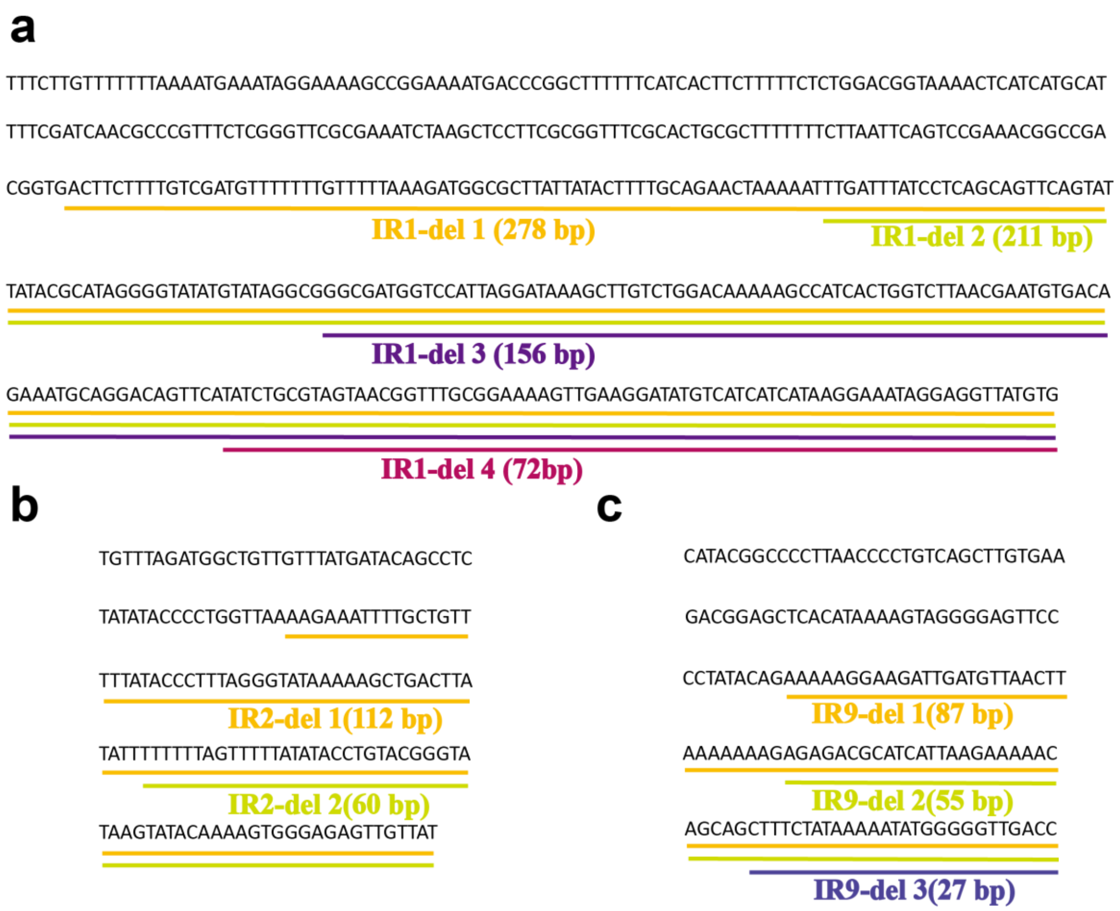

Supplementary Figure 5. The schematic description of truncated fragments in the three intergenic regions. Fragments 1-del 1-4, 2-del 1-2, and 9-del 1-3 were from intergenic regions 1, 2 and 9, respectively. The truncated fragments were underlined with lines in different colors.

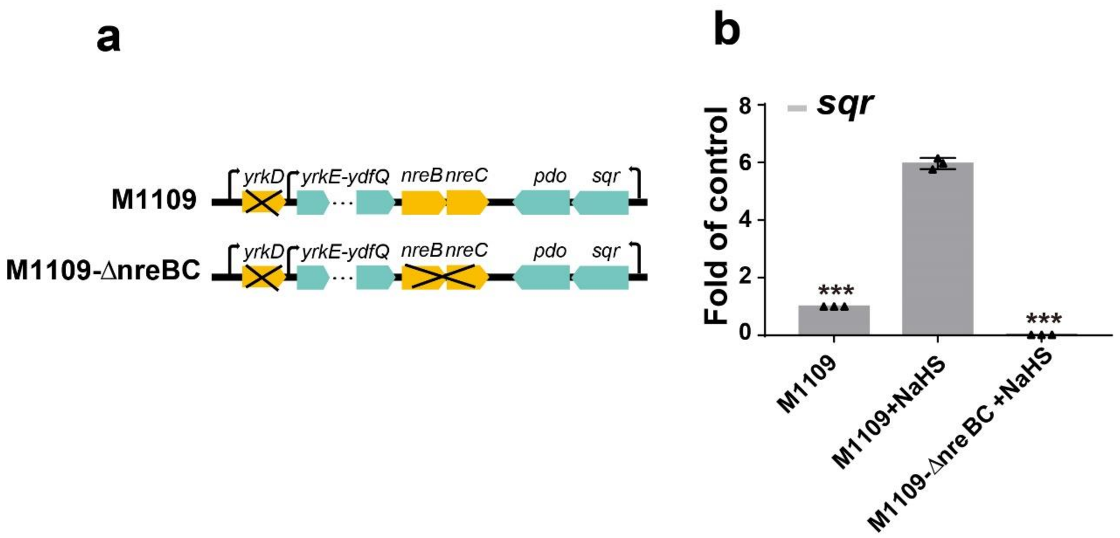

Supplementary Figure 6. The transcription level of *sqr* in M1109 and M1109-Δ*nreBC*. **a)** The schematic graph for the deletion of *nreBC* in M1109. **b)** M1109 and its derived *nreBC* deletion mutant were cultured in the modified M9 medium until their OD_{600} reached at around 0.8. After induction with 200 μM NaHS for 10 min, the transcription level of *sqr* was measured by using the RT-qPCR method, and the *gyrB* was used as a reference gene. Three parallel experiments were performed to obtain the averages and standard deviations. Data are presented as mean ± SD. One-way ANOVA was performed to calculate the p-values, and asterisks indicate a statistically significant difference (***$p < 0.001$). M1109 with H_2S induction (M1109+NaHS) was used as the control

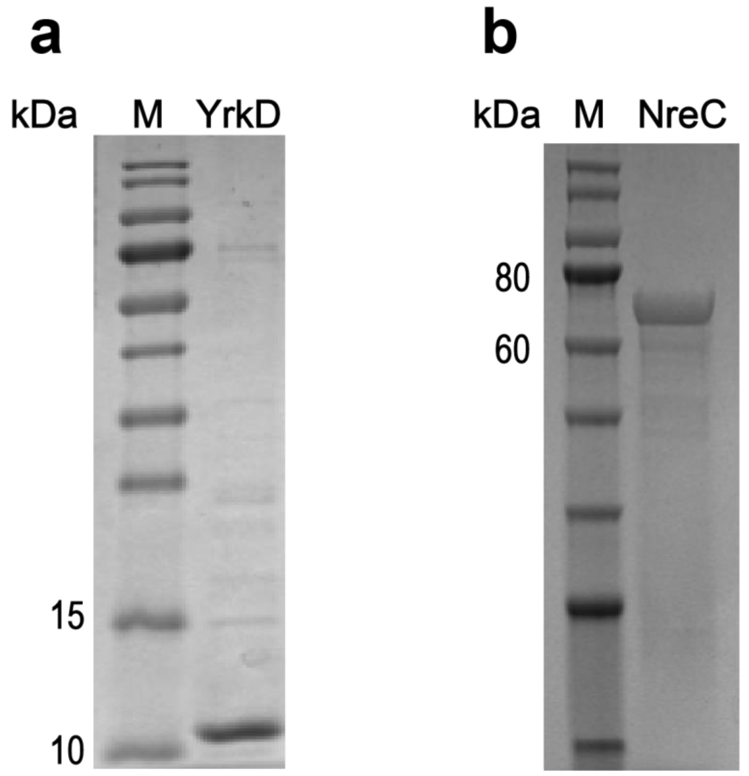

Supplementary Figure 7. Sodium dodecyl sulfate-polyacrylamide gel electrophoresis analysis of the purified proteins. a) His-YrkD (MW is 10857 Da). **b)** MBP-NreC (MW is 66553 Da). M: *Blue Plus* IV Protein Marker (TransGen Biotech)

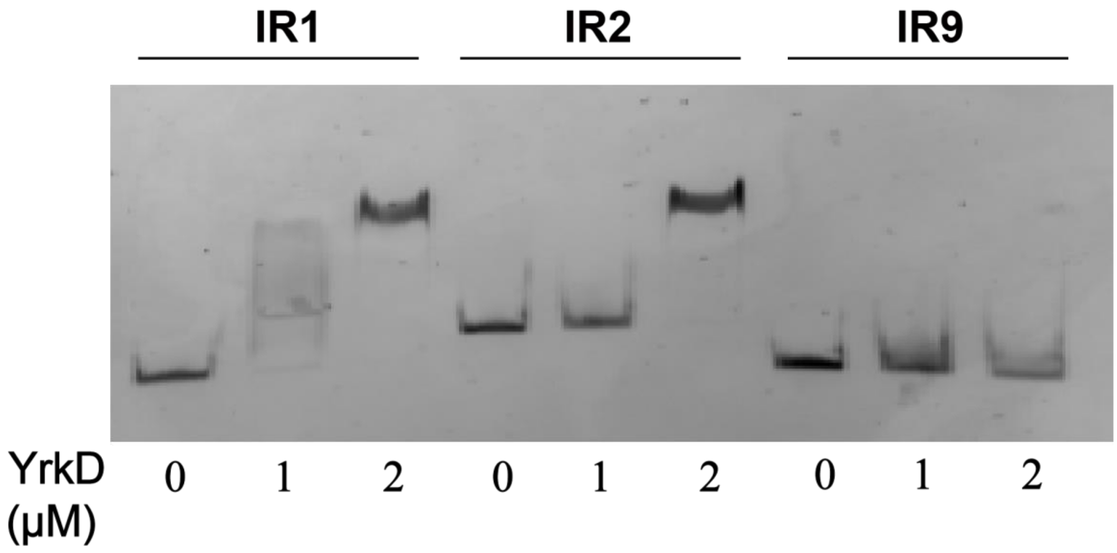

Supplementary Figure 8. The DNA binding properties of YrkD. The binding ability was characterized by using EMSA. 20 nM DNA probes were used. The protein concentrations were indicated in the figure. The binding of YrkD to IR9 with IR1 and IR2 as the positive control.

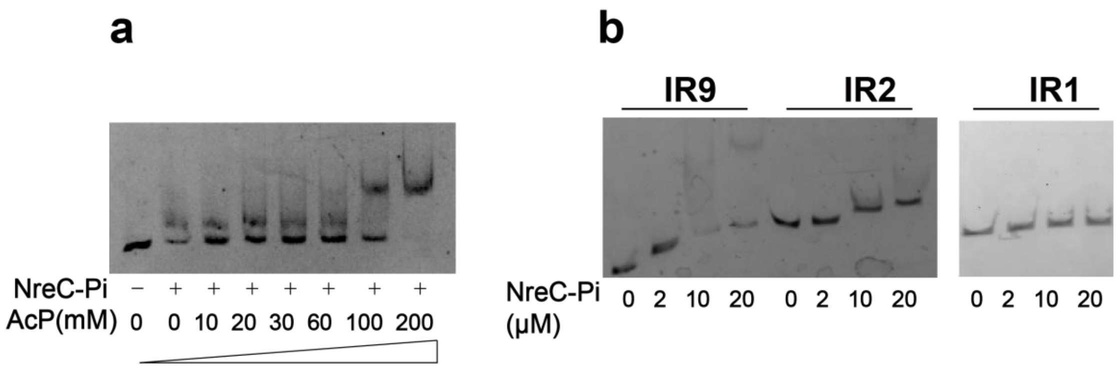

Supplementary Figure 9. The DNA binding properties of NreC. The binding ability was characterized by using EMSA. 20 nM DNA probes were used. **a)** 20 μM NreC was reacted with different concentrations of AcP for 30 min before the reaction. **b)** After NreC was phosphorylated, indicated amount of NreC-Pi was used to test its binding ability with DNA probes. NreC-Pi, NreC was phosphorylated by acetyl-phosphate.

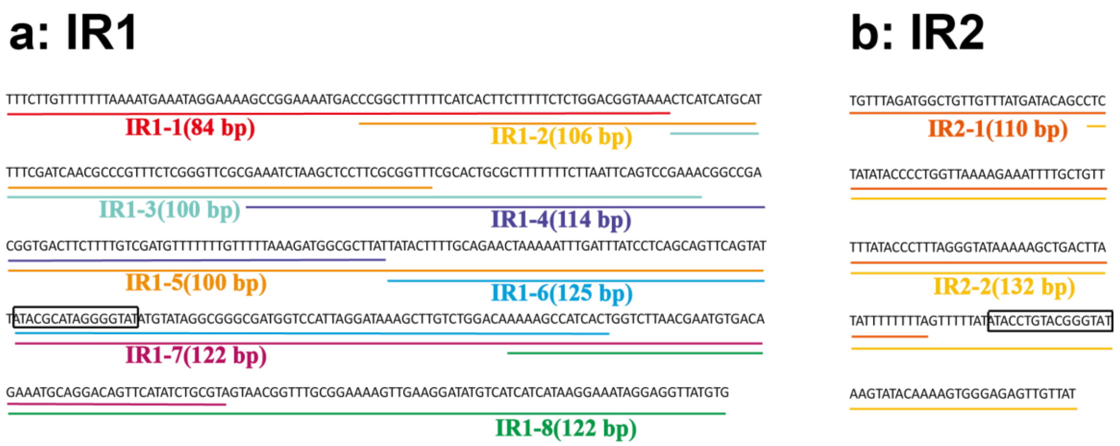

Supplementary Figure 10. The DNA fragments of IR1(a) and IR2(b) were further divided into 8 and 2 fragments to identify the binding sites.

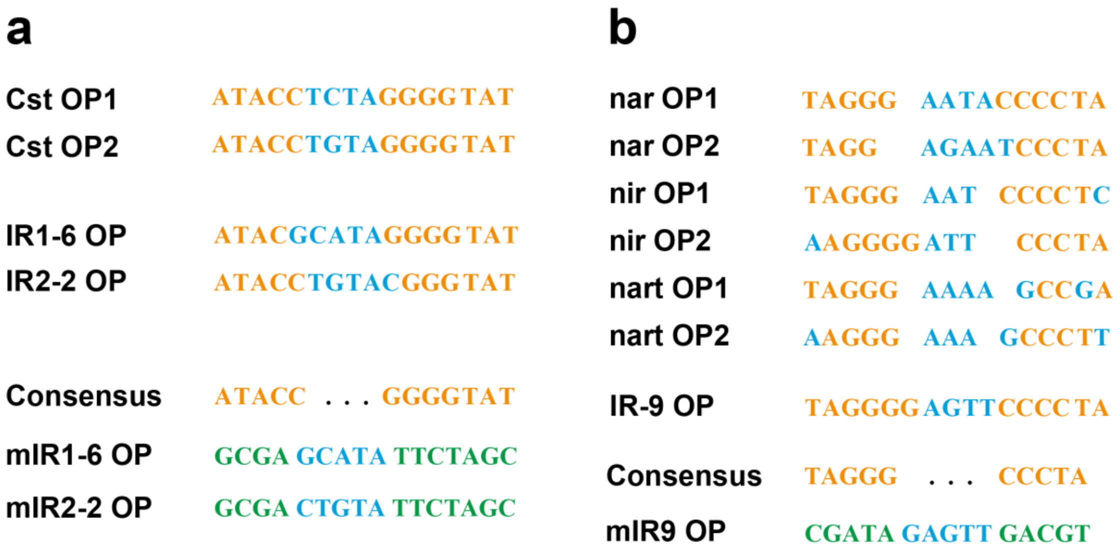

Supplementary Figure 11. The binding sites of YrkD, NreC, and their homologous proteins. a) The binding sites (operators) of *S. aureus* CstR (Cst OP1&2) are compared with those of *B. licheniformis* YrkD (IR1-6&IR2-2) (this work). **b)** The binding sites (operators) of *S. carnosus* NreC (nar OP1&2, nir OP1&2, nart OP1&2) and predicted operator of *B. licheniformis* NreC (IR-9) (this work). OP, operator. The mutated sites in OP of IR1-6, IR2-2 and IR9 were shown in ‘mIR1-9 OP’, ‘mIR2-2 OP’ and ‘mIR9-OP, respectively.

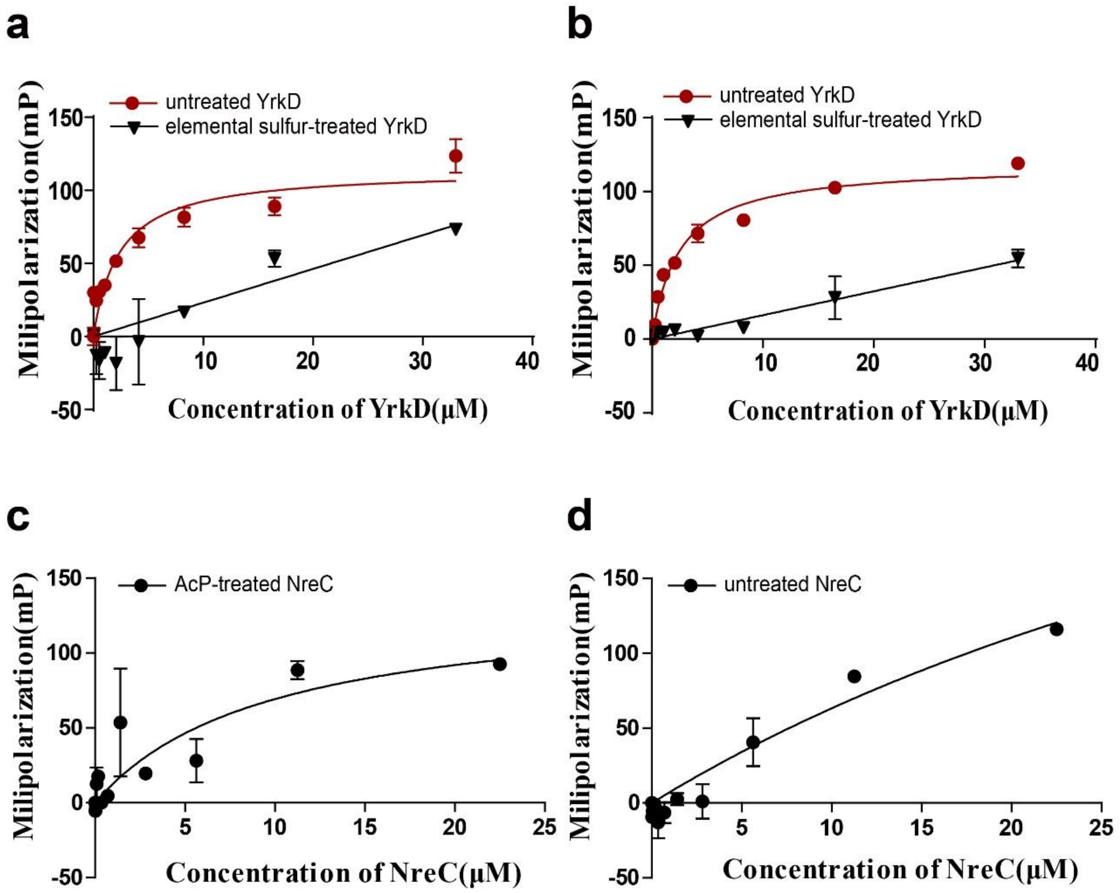

Supplementary Figure 12. The affinity of YrkD and NreC to target DNA probes. **a-b)** YrkD bound to IR1-6 and IR2-2, respectively. The dissociation constants of YrkD to IR1-6 **(a)** and IR2-2 **(b)** were assayed with fluorescence polarization. The black solid triangle indicated the data from elemental sulfur-treated YrkD, and the red solid circle indicated the data from untreated YrkD. **c-d)** NreC bound to IR9. The dissociation constant of NreC to IR9 was assayed with fluorescence polarization (FP). The data from the binding assay of acetate phosphate-treated NreC to IR9 was shown in C, and the data got from the untreated NreC to IR9 was shown in D. In the above experiments, FAM-labeled DNA (1 nM) was incubated with the protein at the indicated concentrations. The reaction solution was treated in the 50 mM Tris-HCl (pH 8.0) at 37°C for 30 minutes in the dark. FP measurements were conducted using

Microplate Reader (BioTek Synergy H1). Three parallel experiments were performed to obtain the averages and standard deviations. Data are presented as mean ± SD. The K_d values were calculated by GraphPad Prism 7 Software (GraphPad).

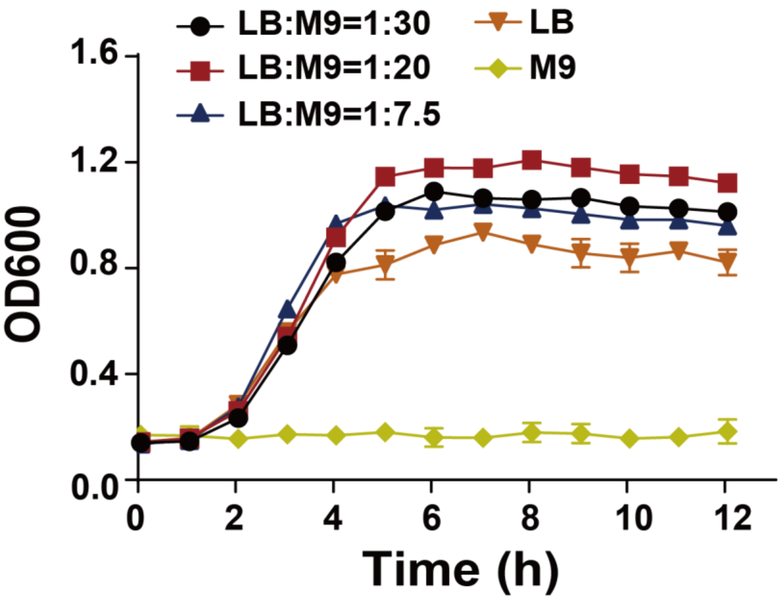

Supplementary Figure 13. The growth of *B. licheniformis* MW3 in different mediums. MW3 was inoculated into mediums at the initial OD_{600} of 0.05 and cultured in a 24-well cell culture plate with continuous shaking at 37°C. The OD_{600} values were recorded at defined time intervals. Three parallel experiments were performed to obtain the averages and standard deviations. Data are presented as mean ± SD.

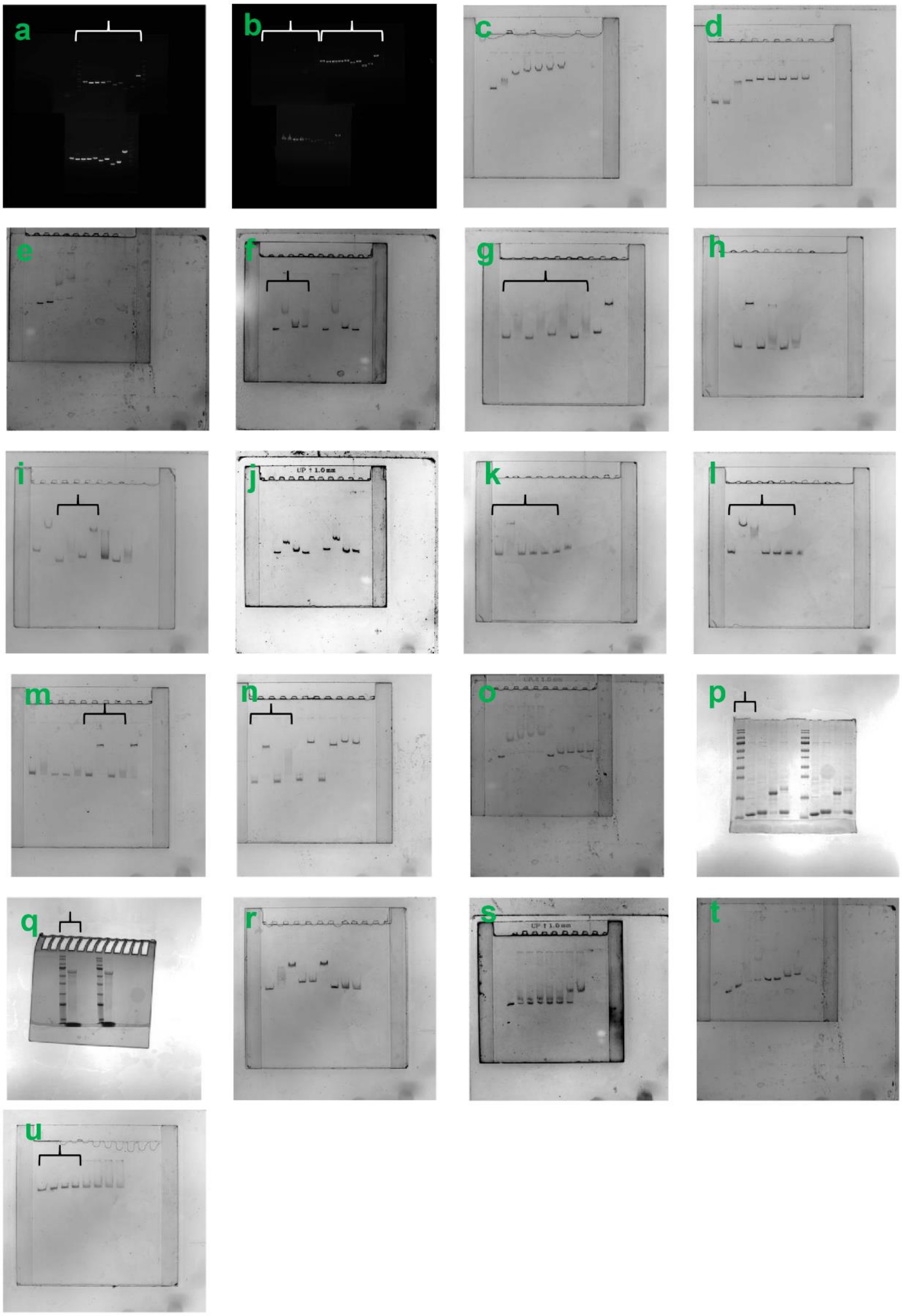

Supplementary Figure 14. Uncropped and unedited gels. a) The uncropped agarose gel of Figure 4a; **b)** The uncropped agarose gel of Supplementary Figure 4a-4b; **c)** The uncropped PAGE gel of Figure 9a; **d)** The uncropped PAGE gel of Figure

9b; **e)** The uncropped PAGE gel of Figure 9c; **f)** The uncropped PAGE gel of Figure 9d; **g-h)** The uncropped PAGE gel of Figure 9e; **i)** The uncropped PAGE gel of Figure 9f; **j)** The uncropped PAGE gel of Figure 9g; **k-l)** The uncropped PAGE gel of Figure 9h; **m-n)** The uncropped PAGE gel of Figure 9i; **o)** The uncropped PAGE gel of Figure 9j; **p)** The uncropped PAGE gel of Supplementary Figure 7a; **q)** The uncropped PAGE gel of Supplementary Figure 7b; **r)** The uncropped PAGE gel of Supplementary Figure 8; **s)** The uncropped PAGE gel of Supplementary Figure 9a; **t-u)** The uncropped PAGE gel of Supplementary Figure 9b;
