# Supplementary material for: A sulfide-sensor and a sulfane sulfur-sensor collectively regulate sulfur-oxidation for feather degradation by Bacillus licheniformis
Source: Commun Biol. 2023 Feb 10;6:167. doi: 10.1038/s42003-023-04538-2 (PMC9918477; doi:10.1038/s42003-023-04538-2)
Supplement: Supplementary file 3 — Description of Additional Supplementary Files [file 42003_2023_4538_MOESM3_ESM.pdf]

Description of Additional Supplementary Files

File name: Supplementary Data 1
Description: Twelve pairs of genes encoding TCSs located near sqr and pdo genes.

File name: Supplementary Data 2
Description: Strains and plasmids used in this study.

File name: Supplementary Data 3
Description: Synthetic oligos used in this study.

File name: Supplementary Data 4
Description: Source data behind the graphs and plots shown in the manuscript.
