# Supplementary material for: A sulfide-sensor and a sulfane sulfur-sensor collectively regulate sulfur-oxidation for feather degradation by Bacillus licheniformis
Source: Commun Biol. 2023 Feb 10;6:167. doi: 10.1038/s42003-023-04538-2 (PMC9918477; doi:10.1038/s42003-023-04538-2)
Supplement: Supplementary file 8 — Reporting Summary [file 42003_2023_4538_MOESM8_ESM.pdf]

nature portfolio

Corresponding author(s): Yongzhen Xia

Last updated by author(s): Jan 15, 2023

Reporting Summary

Nature Portfolio wishes to improve the reproducibility of the work that we publish. This form provides structure for consistency and transparency in reporting. For further information on Nature Portfolio policies, see our Editorial Policies and the Editorial Policy Checklist.

Statistics

For all statistical analyses, confirm that the following items are present in the figure legend, table legend, main text, or Methods section.

n/a	Confirmed	
☐	☒	The exact sample size (*n*) for each experimental group/condition, given as a discrete number and unit of measurement
☐	☒	A statement on whether measurements were taken from distinct samples or whether the same sample was measured repeatedly
☐	☒	The statistical test(s) used AND whether they are one- or two-sided<br>*Only common tests should be described solely by name; describe more complex techniques in the Methods section.*
☒	☐	A description of all covariates tested
☒	☐	A description of any assumptions or corrections, such as tests of normality and adjustment for multiple comparisons
☐	☒	A full description of the statistical parameters including central tendency (e.g. means) or other basic estimates (e.g. regression coefficient) AND variation (e.g. standard deviation) or associated estimates of uncertainty (e.g. confidence intervals)
☒	☐	For null hypothesis testing, the test statistic (e.g. *F*, *t*, *r*) with confidence intervals, effect sizes, degrees of freedom and *P* value noted<br>*Give P values as exact values whenever suitable.*
☒	☐	For Bayesian analysis, information on the choice of priors and Markov chain Monte Carlo settings
☒	☐	For hierarchical and complex designs, identification of the appropriate level for tests and full reporting of outcomes
☒	☐	Estimates of effect sizes (e.g. Cohen's *d*, Pearson's *r*), indicating how they were calculated

Our web collection on statistics for biologists contains articles on many of the points above.

Software and code

Policy information about availability of computer code

Data collection	Data collection from spectrophotometric analysis<br>Hardware: UV-1800 (SHIMADAZU, Japan), software: UV-Probe (Supporting software)<br><br>Data collection from HPLC device<br>LC-20AT (SHIMADAZU, Japan), software: LabSolutions<br><br>Data collection from Real-time quantitative PCR<br>LightCycler 480II instrument (Roche, US), software: Supporting software of this device<br><br>Data collection from fluorescence measurements<br>Microplate spectrophotometer (BioTek Synergy H1, US), software: All-In-One Microplate Reader Software (Supporting software)<br><br>Data collection from fluorescence polarization measurements<br>Hardware: Microplate spectrophotometer (BioTek Synergy H1, US), software: All-In-One Microplate Reader Software (Supporting software)
Data analysis	GraphPad Prism 9.0.0 (Histogram, Dot and line graph, significance analysis, binding saturation-one site specific binding for fluorescence polarization analysis)

For manuscripts utilizing custom algorithms or software that are central to the research but not yet described in published literature, software must be made available to editors and reviewers. We strongly encourage code deposition in a community repository (e.g. GitHub). See the Nature Portfolio guidelines for submitting code & software for further information.

Data

Policy information about availability of data

All manuscripts must include a data availability statement. This statement should provide the following information, where applicable:

- Accession codes, unique identifiers, or web links for publicly available datasets
- A description of any restrictions on data availability
- For clinical datasets or third party data, please ensure that the statement adheres to our policy

All data generated or analysed during this study are included in this published article (and its supplementary information files).

Human research participants

Policy information about studies involving human research participants and Sex and Gender in Research.

Reporting on sex and gender	This information has not been collected.
Population characteristics	See above.
Recruitment	See above.
Ethics oversight	See above.

Note that full information on the approval of the study protocol must also be provided in the manuscript.

Field-specific reporting

Please select the one below that is the best fit for your research. If you are not sure, read the appropriate sections before making your selection.

☒ Life sciences ☐ Behavioural & social sciences ☐ Ecological, evolutionary & environmental sciences

For a reference copy of the document with all sections, see nature.com/documents/nr-reporting-summary-flat.pdf

Life sciences study design

All studies must disclose on these points even when the disclosure is negative.

Sample size	No samples-size calculation was performed. Three parallel samples were required to calculate the statistically significant difference.
Data exclusions	No data was excluded.
Replication	All attempts at replication were successful.
Randomization	The samples (colonies that have the same genotype were treated by the same treatment) were considered as the same group, and the colonies were randomly selected.
Blinding	Blinding was not possible. The genotypes of the strains, as well as the treatments, need to be known before study. The data obtained from these studies are basically supervised by a colleague who is unrelated to the subject. This operation can ensure the reliability of the collected data.

Reporting for specific materials, systems and methods

We require information from authors about some types of materials, experimental systems and methods used in many studies. Here, indicate whether each material, system or method listed is relevant to your study. If you are not sure if a list item applies to your research, read the appropriate section before selecting a response.

Materials & experimental systems

n/a	Involved in the study
☒	☐ Antibodies
☒	☐ Eukaryotic cell lines
☒	☐ Palaeontology and archaeology
☒	☐ Animals and other organisms
☒	☐ Clinical data
☒	☐ Dual use research of concern

Methods

n/a	Involved in the study
☒	☐ ChIP-seq
☒	☐ Flow cytometry
☒	☐ MRI-based neuroimaging
